# Characterization of pubertal development of girls in rural Bangladesh

**DOI:** 10.1371/journal.pone.0247762

**Published:** 2021-04-02

**Authors:** Jinhee Hur, Kerry J. Schulze, Andrew L. Thorne-Lyman, Lee S.-F. Wu, Saijuddin Shaikh, Hasmot Ali, Alain B. Labrique, Keith P. West

**Affiliations:** 1 Center for Human Nutrition, Department of International Health, Johns Hopkins Bloomberg School of Public Health, Baltimore, MD, United States of America; 2 The JiVitA Project, Gaibandha, Bangladesh; BRAC, BANGLADESH

## Abstract

This study aimed to describe the timing and patterns of pubertal maturation of girls living in rural Bangladesh. Starting in September 2015, a total of 15,320 girls from a birth cohort, aged 9 to 15 years at initial encounter, were visited twice at about a one year interval, typically in their birth month. Participants were asked to self-report extent of pubertal maturation, including breast development, pubic hair growth and age at menarche, if applicable. Pubertal stage (abbreviated as B2 and B3-4 for breast development and PH2 and PH3-4 for pubic hair growth) was assigned. Data from both visits were pooled, yielding a total of 29,377 age-related observations per pubertal characteristic. Probit regression models were used to estimate distributions of age at which each stage of pubertal development was attained. Before age 8, <3% of the study population initiated pubertal maturation as indicated by onset of breast development (B2). The median (95% confidence interval) age of B2 and B3-4 was 11.02 (11.00–11.04) and 12.82 (12.80–12.83) years, respectively; and 12.93 (12.91–12.94) and 14.29 (14.27–14.31) years for the onset (PH2) and advanced stage (PH3-4) of pubic hair growth, respectively. Median age at menarche was 13.17 (13.15–13.19) years, with 2.15 years of timespan from B2 to menarche. Girls in rural Bangladesh progressed through puberty following a well-documented sequence of sexual maturation stages. The age at which each pubertal milestone took place was somewhat later, but the tempo from breast development to menarche was comparable to that observed elsewhere. Our findings present a current norm of pubertal maturation in a typical, rural adolescent population in South Asia, which could help inform future studies and interventions to preserve or improve early adolescent health and development.

## Introduction

Adolescence is a life stage during which individuals transition from childhood to adulthood, experiencing physical, physiological and psychological changes. The most salient milestone characterizing adolescence is attainment of reproductive capability through pubertal maturation. South Asia is a region undergoing rapid changes including growing wealth, shifting diets and decreasing physical activity [1]. These changes may influence the timing of sexual maturation of populations in the region [2], as indicated by an estimate of the onset of the primary sex characteristic (i.e. menarche) in rural Bangladesh occurring at age 13.0 years in 2017 [3], compared to age 15.8 years in 1976 [4]. A small number of studies have reported the timing of secondary sex characteristics (e.g. breast development and pubic hair growth) in the region since the late 1980s [5–9], with initial stages of breast development occurring at age ~10 years and pubic hair growth in the months that follow. Given the potential for regional and temporal variation in sexual maturation [10–12] against trends in recent years of generally improving nutritional conditions in historically, chronically undernourished rural Bangladesh [1], there is utility to periodically monitor the timing, sequence and progression of pubertal milestones as an indicator of adolescent maturity and early reproductive health [3, 4, 13, 14].

The timing of puberty in rural South Asian cultures can markedly affect trajectories of socialization, empowerment and agency [15], with menarche commonly viewed to indicate a young girl’s entry into adulthood and readiness for marriage [16, 17]. Coupled with infrequent contraception among newlyweds [18], early marriage can lead to mid-adolescent pregnancy [19], which can adversely affect nutritional status, decelerate growth [20], and thus potentially contribute to endemic short stature among rural South Asian women [20], in addition to often restricting educational achievement of young women [16]. These sequences suggest there is important information to gain by characterizing the timing and progression of pubertal maturation as one approach to monitor health and inform policies intending to delay early marriage and pregnancy, improve nutrition, growth and development, and foster social development among young women in high-risk populations.

In a birth cohort of >15,000 girls aged 9 to 15 years at enrollment living in a large, typical, population-dense, rural setting of Bangladesh, data collected between 2015 and 2017 were used to 1) describe the timing, sequence and progression of prominent features of female sexual maturation, namely menarche, breast development and pubic hair growth, and 2) relate observed patterns of development to those published from other adolescent populations around the world.

## Methods

### Study population

**Fig 1:**
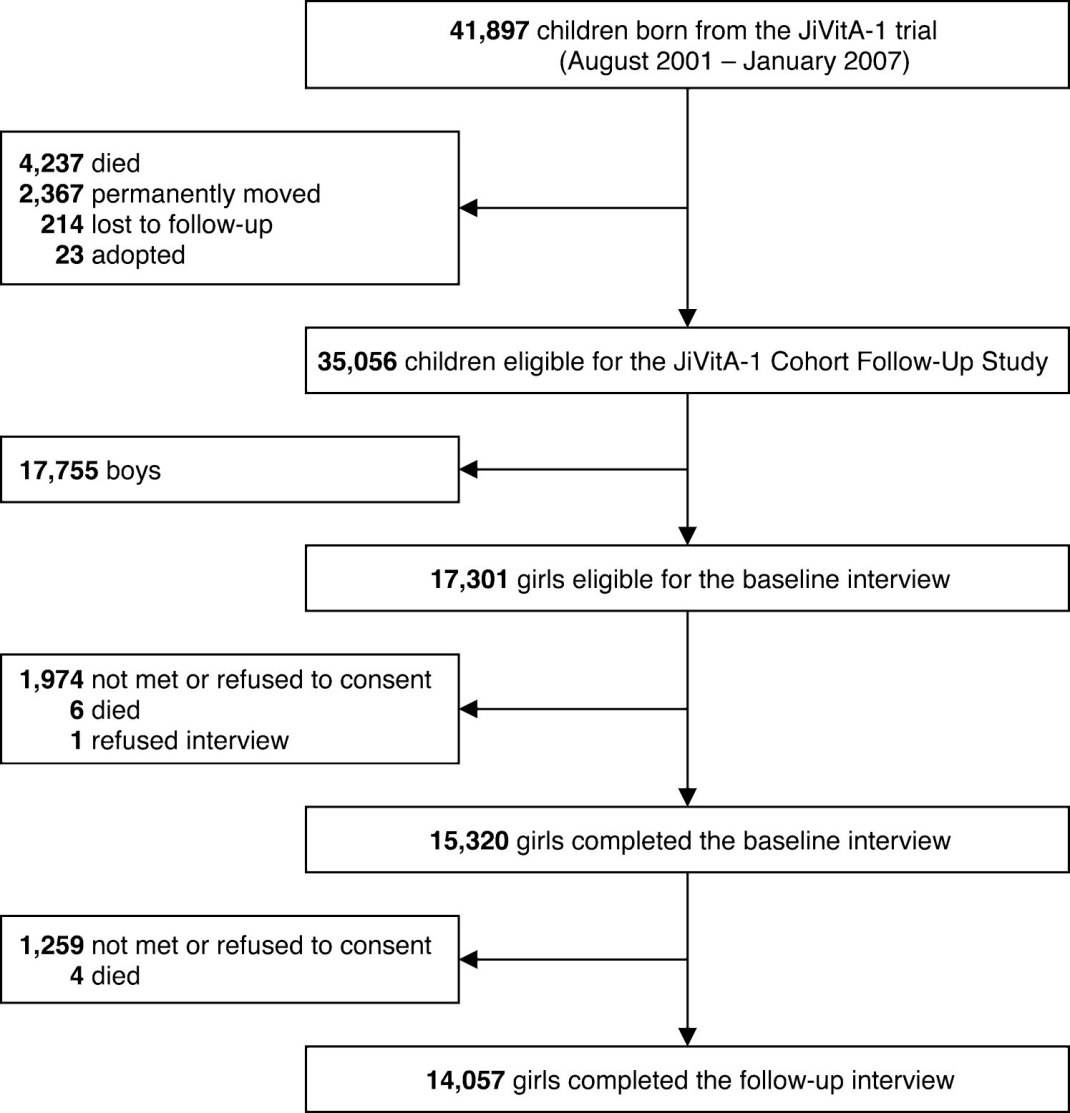
Flow diagram of the study participants.

The JiVitA-1 Cohort Follow-Up Study was a prospective assessment of a birth cohort of 35,056 young adolescents, comprising 17,755 boys and 17,301 girls aged 9 to 15 at enrollment, a relevant age range during which a series of pubertal events take place [21] (Fig 1). The children were born to mothers who participated in a double-masked, cluster-randomized trial of weekly, antenatal vitamin A or β-carotene supplementation, JiVitA-1, conducted in Gaibandha District in northwestern Bangladesh from August 2001 through January 2007 [22]. Gaibandha is an area that reflects typical rural characteristics of Bangladesh [23], a region historically beset by low socioeconomic status [24], children being born low birth weight [25], high infant mortality [26] and chronic undernutrition among preschool children [27] and women in the reproductive years with underlying high levels of food insecurity [28, 29]. Maternal characteristics of the current study population have been described previously [22].

In March 2015, prior to the formal start of the adolescent follow-up study, an initial census was executed to update the vital and residential status of the entire birth cohort. Starting in September 2015, resident children were visited at home during their birth month by trained female interviewers to assess pubertal development, anthropometry, attained level of schooling and household socioeconomic status. With recorded birth dates, exact ages were known on the date of assessment. As the study was designed to assess children during their birth month, the distribution of ages was concentrated around each child’s known birth month across ages 9 to 15 years. The cohort study was designed to follow up children a year later, from ~10 to 16 years in order to assess progressions in growth, maturation, nutritional status and other factors.

In this analysis of female pubertal development, a total of 15,320 adolescent girls aged 9–15 years at the time of 1^st^ interview served as the intended sample, of whom 1,263 were lost to follow-up by the time 2^nd^ visit, leaving 14,057 girls for follow-up assessment by the same cadre of interviewers at a median (interquartile range, 10^th^ and 90^th^ percentiles) of 12.5 (12.0–13.3, 11.8 and 14.6) months later.

### Pubertal development

At both baseline and follow-up visits, trained female staff interviewed girls in the presence of their mother or female guardian, providing an assuring interview setting and opportunity for parental guidance. Pubertal benchmarks were assessed using questions that were adapted from the Pubertal Development Scale (PDS) [30] to the age range and levels of maturation expected of participants, and which included menarche plus secondary sex characteristics of breast development and pubic hair growth for which Tanner stages [31] exist. Following an introduction during which interviewers explained the physical changes that take place during adolescence, girls were asked to report the extent of their breast development and growth of pubic hair. Specifically for pubic hair growth, a pictogram with a mark around the pubic region was shown to girls to facilitate ascertainment. Each girl was asked first whether or not she had experienced each pubertal event. If so, they were probed further about whether the event had recently begun or had been well underway, yielding a three-level ordinal response scale for each secondary sex characteristic: 1) prepubertal (not yet begun); 2) beginning; or 3) in an advanced stage of development. Although the original PDS had a response option for a postpubertal stage [30], it was not included in our adapted questionnaire due to the pubertal age range of participants.

Based on responses from the questionnaire, we categorized girls into maturational stages corresponding to those established by Tanner [31], where scales range successively from stage 1 indicating prematurity to stage 5 representing full maturity of each sex characteristic (Table 1). Girls who reported their breasts had not started to develop were categorized as stage 1 (B1). Stage 2 (B2) was assigned to girls who reported their breasts had just begun to develop. Stage 3–4 (B3-4) was assigned for those who answered their breast development had been well underway. We could not assign stage 5 as we did not ask if breast development was completed. The same classification was applied to gauge maturational progression of pubic hair growth (e.g. PH1, PH2 and PH3-4). We were not able to clinically verify girls’ responses primarily due to the sensitivity surrounding clinical ascertainment of puberty in a rural community setting.

**Table 1:** Classification of maturational stages of breast development and pubic hair growth by Tanner and current study.

Tanner stage	Definition by Tanner^1^	Adapted stage	Response associated with definition in current study^2^
*Breast development*			
B1	Pre-adolescent; elevation of papilla only	B1	Breasts had *not* begun to develop
B2	Breast bud stage; elevation of breast and papilla as a small mound, enlargement of areola diameter	B2	Breasts had *just* begun to develop
B3	Further enlargement of breast and areola with no separation of their contours	B3-4	Breast development was *well underway*
B4	Projection of areola and papilla to form a secondary mound above the level of the breast		
B5	Mature stage; projection of papilla only, due to recession of the areola to the general contour of the breast		Not applicable
*Pubic hair growth*			
PH1	Pre-adolescent; the vellus over the pubes is not further developed than that over the anterior abdominal wall, i.e. no pubic hair	PH1	Pubic hair had *not* begun to grow
PH2	Sparse growth of long, slightly pigmented, downy hair, straight or only slightly curled, appearing chiefly along the labia	PH2	Pubic hair had *just* begun to grow
PH3	Considerably darker, coarser and more curled. The hair spreads sparsely over the junction of the pubes	PH3-4	Pubic hair growth was *well underway*
PH4	Hair is now adult in type, but the area covered by it is still considerably smaller than in most adults. There is no spread to the medial surface of the thigh		
PH5	Adult in quantity and type, distributed as an inverse triangle of the classically feminine pattern. Spread to the medial surface of the thighs, but not up the linea alba or elsewhere above the base of the inverse triangle		Not applicable

^1^ Derived from Tanner [31].

^2^ Adapted version of the Pubertal Development Scale (PDS), developed by Petersen et al [30], was used in our questionnaire.

For menarche, girls were asked to recall if they had experienced a first menstrual cycle and, if so, in what calendar year and month. To calculate menarcheal age, the default calendar day of 15 was assigned. If girls had reportedly attained menarche in the same month as when they were interviewed, a calendar day of 1 was assigned. For those who were premenarcheal at the baseline interview but reported to have attained menarche within the same month, as recalled at the follow-up assessment a year later, the last day of the month was assigned.

### Statistical analysis

The distributions of stages of breast development and pubic hair growth by age are presented according to the 1^st^ or 2^nd^ visit and as total observations merged from both visits. Girls were grouped by age in years using mid-year as a cutoff; for example, girls aged 9.50–10.49 were considered to be 10-year-olds. Ages at stages of breast development and pubic hair growth were summarized as calculated means ± standard deviations (SDs). To maximize the age range presented and accommodate potential temporality in pubertal development, we pooled the data collected at baseline and follow-up assessments, with girls therefore contributing up to two maturational and age time points, yielding a total of 29,377 observations.

The prevalence of menarche and different maturational stages of breast development and pubic hair growth was calculated for each year of age at assessment. For breast development and pubic hair growth, proportions of girls at each age having achieved maturational stage 2 or greater (i.e. B2 or B3-4; PH2 or PH3-4) and stage 3 or greater (i.e. B3-4; PH3-4) were computed, respectively.

Probit regression was used to plot cumulative frequency curves for different stages of pubertal maturation according to age at assessment. In brief, a probit model fits the probability of the event, which follows an inverse standard normal distribution, as a form of linear regression with predictors of interest [32]. In our analysis, separate models were fit for different maturational stages of pubertal events with age in years as the independent variable of interest.

We next estimated ages at median (50^th^) and selective (3^rd^, 10^th^, 25^th^, 75^th^, 90^th^ and 97^th^) percentiles of the onset of menarche and different maturational stages of breast development and pubic hair growth. A median age was obtained when the predicted probability from the probit model equaled 0.5, indicating the estimated age at which half of the study population reached a given maturational stage. As a supplementary analysis, we performed separate probit analysis for the data collected at baseline versus follow-up assessment. All statistical analyses and calculation were conducted using STATA 14 (StataCorp LP, College Station, Texas, USA).

### Ethical review and approval

Ahead of study enrollment, all participating adolescent girls gave verbal assent with parental or guardian’s written consent. Both the maternal trial [22] and current adolescent follow-up study were reviewed and approved by the institutional review board at the Johns Hopkins Bloomberg School of Public Health, Baltimore, MD and the Bangladesh Medical Research Council, Dhaka, Bangladesh.

## Results

A total of 15,320 and 14,057 girls were eligible at baseline and follow-up assessments, respectively, contributing a total of 29,377 analyzed observations. At baseline, 38.3% of girls had a height-for-age z-score <-2 [33] and 25.5% had a body mass index-for-age z-score <-2 [33] (S1 Table). Nearly all girls (96.3%) were currently enrolled in school. Nutritional and socioeconomic status of 1,263 girls who were lost to follow-up was largely comparable to those who remained in the study.

The age distributions of participants at baseline and follow-up visits are presented in Table 2 according to 1-year age strata and maturational stages of breast development and pubic hair growth. The age range at baseline was 9–15 years with few (0.1%) 15-year-olds; whereas by the follow-up visit 11.3% of girls had aged to 15 years or older. Maturational stages for each secondary sex characteristic expectedly advanced with age at the follow-up visit. For example, initially 31.5% of girls were in the earliest stage of prepubertal breast development (B1), which declined to 12.6% at follow-up. Pubic hair was inapparent (PH1) in 71.0% of girls at baseline, which declined to 45.3%. Age distributions within each 1-year interval are older by ~0.1 year at the follow-up visit, an artifact of imbalance across age intervals, and likely explaining the older follow-up age distributions within each stage of breast development and public hair growth. Consequently, Table 2 also presents composite mean ± SD ages for the two visits combined within each age stratum and maturational stage in order to present average sexual maturation patterns for this population, including those for 9- and 16-year-old children, which are uniquely available from the baseline and follow-up assessments, respectively.

**Table 2:** Distributions of age and maturational stages of breast development and pubic hair growth at baseline and follow-up visits of adolescent girls in rural Bangladesh.

	At baseline		At follow-up		Combined visits	
	*n* (%)	Age, years (Mean ± SD)	*n* (%)	Age, years (Mean ± SD)	*N* (%)^1^	Age, years (Mean ± SD)^2^
Age, years						
9	189 (1.2)	9.1 ± 0.1			189 (0.6)	9.1 ± 0.1
10	2,507 (16.4)	10.0 ± 0.1	177 (1.3)	10.1 ± 0.1	2,684 (9.1)	10.0 ± 0.1
11	2,983 (19.5)	11.0 ± 0.1	2,302 (16.4)	11.1 ± 0.1	5,285 (18.0)	11.1 ± 0.1
12	3,698 (24.1)	12.0 ± 0.1	2,734 (19.5)	12.1 ± 0.1	6,432 (21.9)	12.1 ± 0.1
13	4,259 (27.8)	13.0 ± 0.1	3,416 (24.3)	13.1 ± 0.1	7,675 (26.1)	13.1 ± 0.1
14	1,669 (10.9)	14.0 ± 0.1	3,848 (27.4)	14.1 ± 0.1	5,517 (18.8)	14.1 ± 0.1
15	15 (0.1)	14.6 ± 0.1	1,559 (11.1)	15.0 ± 0.2	1,574 (5.4)	15.0 ± 0.2
16			21 (0.2)	15.6 ± 0.1	21 (0.1)	15.6 ± 0.1
Total	15,320 (100.0)	12.0 ± 1.3	14,057 (100.0)	13.0 ± 1.3	29,377 (100.0)	12.5 ± 1.4
Maturation						
Breast development, stage						
B1	4,821 (31.5)	10.8 ± 1.0	1,777 (12.6)	11.5 ± 0.8	6,598 (22.5)	11.0 ± 1.0
B2	5,690 (37.1)	12.1 ± 1.0	4,306 (30.6)	12.4 ± 1.1	9,996 (34.0)	12.2 ± 1.1
B3-4	4,809 (31.4)	13.0 ± 0.9	7,974 (56.7)	13.7 ± 1.0	12,783 (43.5)	13.5 ± 1.0
Total	15,320 (100.0)		14,057 (100.0)		29,377 (100.0)	
Pubic hair growth, stage^3^						
PH1	10,871 (71.0)	11.5 ± 1.2	6,368 (45.3)	12.2 ± 1.1	17,239 (58.7)	11.8 ± 1.2
	3,134 (20.5)	12.9 ± 0.9	3,750 (26.7)	13.4 ± 1.0	6,884 (23.5)	13.2 ± 1.0
PH3-4	1,305 (8.5)	13.3 ± 0.8	3,928 (28.0)	14.1 ± 0.9	5,233 (17.8)	13.9 ± 0.9
Total	15,310 (100.0)		14,046 (100.0)		29,356 (100.0)	

Abbreviations: B1, stage 1 for breast development; B2, stage 2 for breast development; B3-4, stage 3–4 for breast development [30]; PH1, stage 1 for pubic hair growth; PH2, stage 2 for pubic hair growth; PH3-4, stage 3–4 for pubic hair growth [30]; SD, standard deviation

^1^ Represents the total number (%) within each age and maturational stage across both visits.

^2^ Represents the unadjusted mean of stratum-specific ages from both visits combined.

^3^ Girls who refused to answer at baseline (*n* = 10) and follow-up (*n* = 11) were excluded.

**Fig 2:**
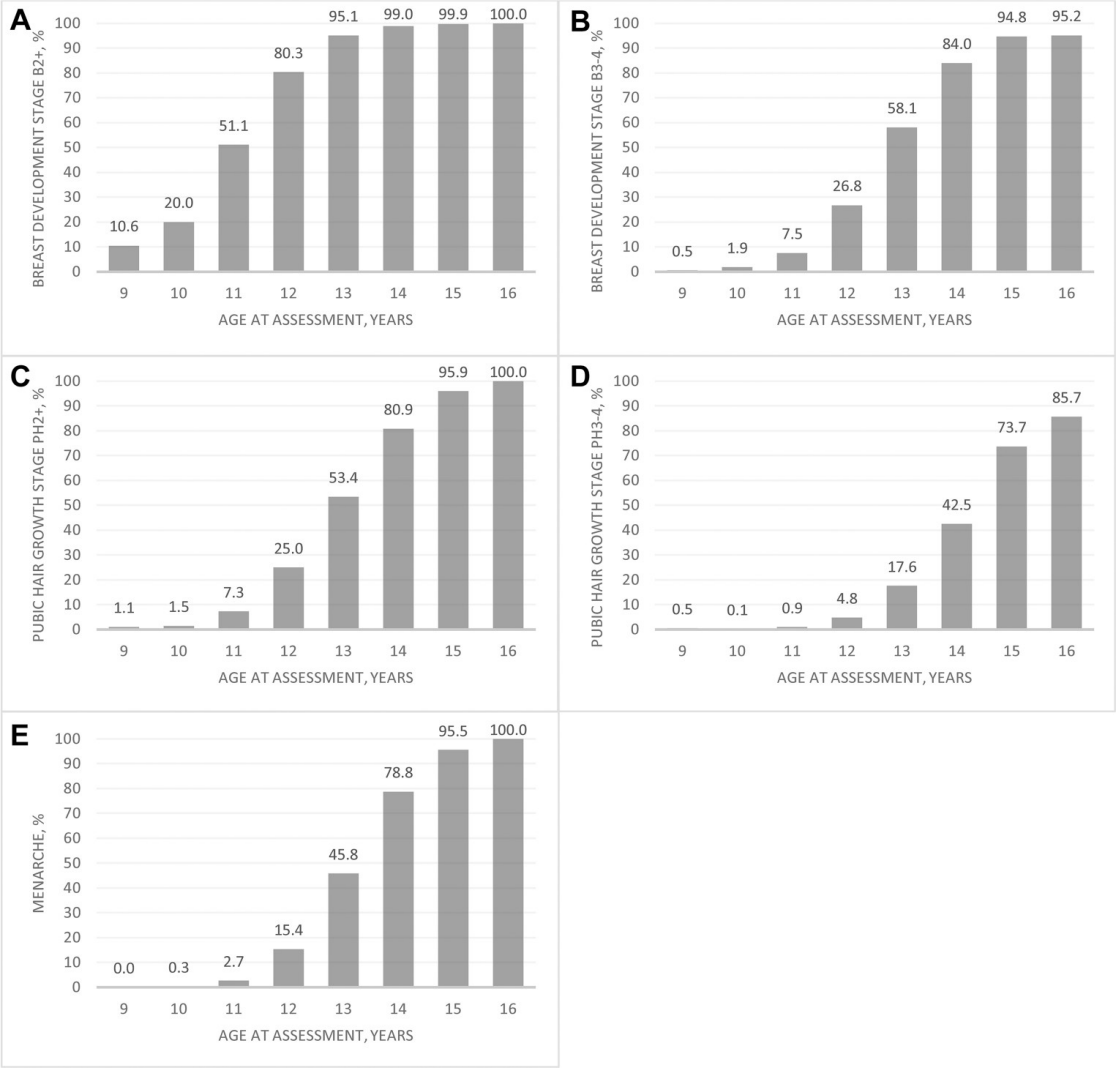
Prevalence of pubertal maturational stages by age at assessment of adolescent girls in rural Bangladesh. A, Percent attainment of breast development stages B2+ and, B, B3-4. C, Percent attainment of pubic hair growth stages PH2+ and, D, PH3-4. E, Prevalence of menarche. All ages are centered on birth dates ± 6 months.

Fig 2 summarizes composite age-specific patterns of attained breast development and pubic hair growth (for stages 2+ and 3–4) and menarche. At age 9 (i.e. an interval encompassing 8.50–9.49 years), 10.6% reported onset of breast development (B2+), with the percent rising steeply with age such that by 14 (i.e. 13.50–14.49 years) virtually all children had begun breast development (Fig 2A), and by age 15 years 94.8% were in an advanced stage (B3-4) (Fig 2B). On the other hand, pubic hair growth lagged, evident by <2% of 9–10 (i.e. 8.50–10.49) year-olds reporting any growth (PH2+) and a slower rise in percent attaining this characteristic (Fig 2C). At 15 and 16 years, 95.9% and 100% of girls, respectively, reported having pubic hair, and 73.7% and 85.7%, respectively, in an advanced stage of hair growth (Fig 2D). Menarche lagged both secondary characteristics, with 2.7% of aged 11 (i.e. 10.50–11.49 years) and 15.4%, 45.8% and 78.8% of girls becoming postmenarcheal at average exact ages of 12, 13 and 14, respectively. Virtually all girls were postmenarcheal by age 16 (i.e. 15.50–16.49) years (Fig 2E).

**Fig 3:**
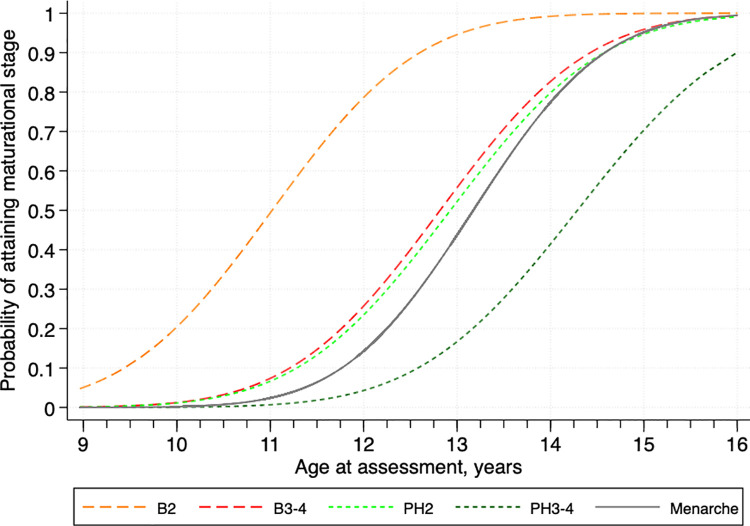
Cumulative frequency curves for pubertal development of adolescent girls in rural Bangladesh. Abbreviations: B2, stage 2 for breast development; B3-4, stage 3–4 for breast development; PH2, stage 2 for pubic hair growth; PH3-4, stage 3–4 for pubic hair growth. Patterns represent composite of two assessments ~1 year apart. All ages are centered on birth dates ± 6 months.

Fig 3 demonstrates the cumulative, composite frequency curves plotted from a probit model revealing intervals between maturational stages of each pubertal milestone by age. We note that, on average, the onset of breast development initiates puberty, followed by pubic hair growth and menarche, with all except the advanced stage of pubic hair growth converging asymptotically by age 16 (i.e. 15.50–16.49) years.

Complementing Fig 2, the age distributions in attained stages 2 and 3–4 of secondary sex characteristics and menarche, estimated from the probit model, are presented in Table 3. The median (95% confidence interval [CI]) age of onset of breast development (B2) was 11.02 (11.00–11.04) years. For the onset of pubic hair growth (PH2) and menarche, the median (95% CI) age was 12.93 (12.91–12.94) and 13.17 (13.15–13.19) years, respectively. The duration of overall pubertal progression, often designated as the interval between age at breast development and menarche, was 2.15 years. The median intervals from B2 to other stages of pubertal milestones were 1.80, 1.91 and 3.27 years for B3-4, PH2 and PH3-4, respectively. As the data presented in Fig 2 and Table 3 summarize the composite data, distributions of the timing, sequence and progression of attainment of each pubertal milestone are also presented in supplementary material, for baseline (S1 Fig and S2 Table) and follow-up (S2 Fig and S3 Table) assessments, showing that they mirror one another, differing only to minor degrees, and thus reinforce the representativeness of the more encompassing composite distributions of sexual maturation.

**Table 3:** Ages at which different stages of secondary sex characteristics and menarche were attained^1^.

Maturation	Age, years						
	P_3_	P_10_	P_25_	P_50_ (95% CI)	P_75_	P_90_	P_97_
Breast development, stage							
B2	8.70	9.44	10.19	11.02 (11.00–11.04)	11.85	12.60	13.34
B3-4	10.46	11.21	11.97	12.82 (12.80–12.83)	13.66	14.43	15.18
Pubic hair growth, stage							
PH2	10.51	11.28	12.06	12.93 (12.91–12.94)	13.79	14.57	15.34
PH3-4	11.78	12.58	13.39	14.29 (14.27–14.31)	15.19	16.00	16.79
Menarche	11.11	11.76	12.43	13.17 (13.15–13.19)	13.91	14.58	15.24

Abbreviations: B2, stage 2 for breast development; B3-4, stage 3–4 for breast development; CI, confidence interval; P_3-97_, 3^rd^-97^th^ percentile, respectively; PH2, stage 2 for pubic hair growth; PH3-4, stage 3–4 for pubic hair growth.

^1^ Ages at attainment were estimated from probit analysis.

## Discussion

In a large, contemporary birth cohort of young adolescent girls living in a typical rural setting in northwestern Bangladesh, we have described the sequence and tempo of pubertal development, reflected by breast development, pubic hair growth and menarche, for which international references exist for comparison. We observed the onset of these three events to occur, on average, at ages 11.02, 12.93 and 13.17 years.

The timing of the onset of breast development and menarche was found to be comparable to what has been reported from a well-published British population studied in the 1960s in which these pubertal milestones occurred at ages 11.15 and 13.47 years, respectively [21]. We have also placed our findings in context of relevant studies in the literature since the late 1970s, prioritized by their multiple pubertal milestones estimated with the use of probit analysis (Table 4). Also relevant, are findings from a recent meta-analysis of 27 studies in adolescent populations born in or after 1998 in low and middle income countries (LMICs) that reported pooled mean ages of 10.4 and 12.3 years for breast development and menarche, respectively [2]. From these comparisons, rural Bangladeshi girls appear to be maturing later than other adolescent populations in LMICs. Of note, 19 out of 27 studies included in the meta-analysis came from urban settings, which could affect developmental timing. Also, a later menarche was observed in studies from African than Asian countries, at ages 13.8 versus 12.3 years, respectively [2]. Together, these studies reveal variation in pubertal timing by country over a period of five decades within LMICs and that pubertal timing in our study population plausibly reflects later pubertal development in rural contexts. Beyond potential differences associated with study design, inter-population and decadal variation may be explained by suboptimal nutritional and socioeconomic status that often exist in rural Bangladesh [3, 34].

**Table 4:** Mean or median age at the onset of secondary sex characteristics reported from different populations.

Author	Study period	Country	(Subgroup)^1^	Age at sexual maturation, years			Interval of B2– menarche, years	Analytic approach^2^
				B2	PH2	Menarche		
Hur (current study)	2015–2017	Bangladesh		11.02	12.93	13.17	2.15	Probit
*Countries of high income*								
Biro [35]	1986–1996	US	(NHW)	10.44	10.40	12.60	2.16	Prospective
			(AA)	9.78	9.68	12.00	2.22	
Wu [36]	1988–1994	US	(NHW)	10.3	10.6	12.6	2.3	Probit
			(AA)	9.5	9.5	12.2	2.7	
			(MA)	9.7	10.3	12.2	2.5	
Juul [37]	1991–1993	Denmark		10.88	11.29	13.42	2.54	Probit
Herman-Giddens [38]	1992–1993	US	(NHW)	9.96	10.51	12.88	2.92	Probit
			(AA)	8.87	8.78	12.16	3.29	
Huen [39]	1993	Hong Kong		9.78	11.64	12.38	2.6	Probit
Miller [40]	1999–2006	US		—	—	12.60	—	Retrospective
Biro [41]	2004–2011	US	(NHW)	9.7	—	—	—	Prospective
			(AA)	8.8				
			(Hispanic)	9.3				
			(Asian)	9.7				
Aksglaede [42]	2006–2008	Denmark		9.86	11.09	13.13	3.27	Probit
*Countries of low and middle income*								
Chowdhury [4]	1976	Bangladesh		—	—	15.8	—	Probit
Qamra [7]	1980s	India	(HSES)	9.9	10.3	12.0	2.1	Probit
			(LSES)	10.6	11.1	12.8	2.2	
Agarwal [5]	1988–1991	India	(HSES)	10.2	—	12.6	2.4	Probit
Rao [43]	1990s	India	(HSES)	—	—	12.1	—	Probit
			(LSES)			15.4		
Chowdhury [13]	1996	Bangladesh		—	—	13.0	—	Probit
Mahachoklertwattana [44]	1997–1999	Thailand		9.4	11.1	11.2	1.8	Probit
Rabbani [45]	2001–2004	Iran		10.15	10.48	14.54	4.39	Probit
Ma [46]	2003–2005	China		9.20	11.16	12.27	3.07	Probit
Kashani [47]	2005–2006	Iran		10.14	10.78	12.65	2.51	Probit
Dasgupta [48]	2005–2011	India		—	—	11.57	—	Probit
Opare-Addo [49]	2008	Ghana		11.10	11.20	12.89	1.79	Probit, Retrospective
Khadgawat [8]	2013	India	(HSES)	10.8	10.9	12.4	1.6	Probit
Rahmawati [50]	2015	Indonesia	(HSES)	—	—	12.01	—	Retrospective
			(LSES)			12.97		

Abbreviations: AA, African American; B2, stage 2 for breast development; HSES, high socioeconomic status; LSES, low socioeconomic status; MA, Mexican American; NHW, non-Hispanic white; PH2, stage 2 for pubic hair growth.

^1^ Subgroup was indicated as if the original data were presented.

^2^ Analytic approach used by the authors of each study to estimate ages at reaching pubertal milestones was classified into three: retro- and prospective methods and probit analysis. Retrospective approach estimates a simple, descriptive statistic (i.e. a mean or median) of age at puberty using the recalled data from individuals. Prospective approach records the onset of puberty in a timely manner through a regular follow-up of prepubertal children. Probit analysis estimates age at puberty using the current state of pubertal maturation (i.e. yes or no) and age at assessment from both pre- and postpubertal adolescents.

Our findings also allow trends in age at menarche (AAM) to be examined over time within rural Bangladesh. In 1976, five years following independence and two years after a major flood and subsequent famine, menarche was reported to be occurring at age 15.8 in Matlab Thana in southeast Bangladesh [4]. By 1996, a study in nearby Narayanganj District reported a mean AAM of 13.0 years [13], which is comparable to our current estimate of 13.2 years in Gaibandha District in the northwest region. Data from our field site collected a decade before the current study reported a mean AAM of 12.8 years [14]. This was derived from an adolescent population in which 88% of girls had attained menarche; thus, by excluding premenarcheal adolescents, the attained estimate could have been biased towards an earlier age. A study conducted between 2016 and 2017 in Matlab Thana reported a median AAM of 13.0 years [3]. Taken together, our findings suggest that menarche has stabilized at ~13 years of age in rural Bangladesh over the last 2 decades. As a downward secular trend in AAM is considered to have plateaued at ~12 years of age as of the 1970s in industrialized countries, a further lowering in AAM may still occur in LMICs with improved living standards and nutritional conditions [10, 51], consistent with an earlier age of breast development being observed around the world [12]. Little data are available to be able to compare trends in pubic hair development within populations over time.

Girls in rural Bangladesh progressed through puberty evident by a predictable sequence of breast development, followed by pubic hair growth on average 1.91 years later, and after another 0.24 years by menarche [31]. A much shorter interval of 0.30–0.55 years between breast development and pubic hair growth, calculated from probit analysis, has been reported from the US and Denmark (Table 4) [36–38]. On the other hand, studies in Thailand [44] and China [39, 46] that used the same statistical approach as ours have reported intervals between 1.70–1.96 years, similar to this study. Whether this variation can be explained by differences in biological phenotype, such as a lack of pubic hair follicles, or production and circulating levels of adrenal androgens [39], remains to be elucidated.

Our observations of the timing of pubertal onset and its distribution, indicated by stage 2 of breast development (B2), were similar to what Marshall and Tanner observed in a longitudinal study of British girls reported in the 1960s [21]. Their pioneering data has evolved to become the standard for assessing extent and progression of pubertal development, including providing a definition of precocious puberty as starting at age 8 or younger, usually assessed by onset of breast development [21]. We estimate that <3% of girls in our study population experienced breast development before age 8.

The interval between ages at breast development and menarche is considered a reliable indicator of the duration of puberty [21]. In our study population, despite a relatively late pubertal onset at 11.02 years, girls progressed from breast development stage B2 to menarche over a period of 2.15 years, comparable to that reported in other populations [7, 35]. In the UK, Marshall and Tanner reported a similar pubertal duration between early versus late maturing girls [21], and more recent studies from Europe have reported that later onset of puberty was followed by a shorter duration of maturation [52, 53]. Of note, the interval between consecutive pubertal events derived from cross-sectional data is likely to appear longer than that from longitudinal data, due to higher variance in estimates between individuals [46].

To our knowledge, this is the first study concurrently characterizing multiple pubertal milestones of girls in rural Bangladesh and comparing findings with those in studied adolescent populations across different regions of the world. With the use of probit analysis, we were able to estimate pubertal timing of the entire, large sample of young adolescents, without biasing our estimates that can result from restricting analysis to those who have attained a pubertal milestone, and improve comparability with other studies that employed probit analysis [4, 5, 7, 8, 13, 36–39, 42–49]. Given the present study comprises a birth cohort with dates of birth known, girls’ ages were calculated accurately to the year, month and day.

As a limitation, rather than being ascertained via clinical examination, pubertal maturation was assessed by lay interviewers administering a questionnaire adapted from the Pubertal Development Scale (PDS) [30], with drawn anatomic site on pubic region to help children answer the questionnaire without showing explicit maturational images. This approach allowed pubertal maturation to be assessed in a very large number of children in a culturally sensitive and acceptable way. Several studies have noted a moderate to high level of agreement (e.g. weighted kappa or Kendall tau of 0.57 to 0.73) between the PDS and clinically ascertained Tanner stages, with greater agreement when the PDS was mapped onto the three-level pubertal scales [54–57], as used in the current study. Of relevance, a recent study of similarly aged and nourished girls in rural Pakistan [57] reported excellent agreement (weighted kappa of 0.73) between the PDS and physician-assessed Tanner stages, supporting the clinical validity of this self-reported method to gauge pubertal maturation in large-scale epidemiologic studies. Our observations of increasing maturation of secondary sex characteristics with advancing age and sequential timing of pubertal milestones were consistent with a well-established pattern of pubertal development [21]. Only ~2% of children reportedly “regressed” in recording breast development and pubic hair growth stages from baseline to the follow-up assessment, while 44% and 40% advanced, and 54% and 57% remained at the same reported stage, respectively, offering evidence of reliability (data not shown). In turn, menarche is a salient and thus particularly memorable event across a series of pubertal milestones; however, a short interval between an interview and past menarche is likely to yield more accurate data on recalled AAM [58]. Among menarcheal girls in our study population, the mean interval between the date of menarche and interview was 6.4 months, which we believe lies within the valid time period to support the accuracy of self-reported AAM [3, 58].

By characterizing multiple pubertal milestones including both primary and secondary sex characteristics in a large, contemporary birth cohort of adolescent girls in rural Bangladesh, the current study contributes knowledge to the literature on pubertal maturation of girls in South Asia [3–9, 13, 43, 44, 50]. Additional evidence on great detail of pubertal milestones of adolescent populations in LMICs, particularly in rural settings, will help establish the generalizability of our findings to other populations. Future studies elucidating nutritional, socioeconomic or environmental determinants of puberty in this context can be expected to contribute to a better understanding of maturational patterns of adolescent girls in South Asia and help inform the designs of interventions intended to benefit their growth, health and development.

In conclusion, adolescent girls in rural Bangladesh progressed through puberty following a well-documented sequence of breast development, pubic hair growth and menarche. The timing and pace of reaching these pubertal milestones appear to be similar to that reported in the western population in the 1960s. Observed onsets of phenotypical pubertal milestones were among the latest in a limited set of studies globally; whereas the tempo for the overall pubertal progression, that is from breast development to menarche, was in a comparable range.

## Supporting information

S1 Checklist STROBE statement—Checklist of items that should be included in reports of *cohort studies*. (DOCX)

S1 Fig Cumulative frequency curves for pubertal development of adolescent girls in rural Bangladesh using data collected at baseline visit. Abbreviations: B2, stage 2 for breast development; B3-4, stage 3–4 for breast development; PH2, stage 2 for pubic hair growth; PH3-4, stage 3–4 for pubic hair growth. All ages are centered on birth dates ± 6 months.
(TIF)

S2 Fig Cumulative frequency curves for pubertal development of adolescent girls in rural Bangladesh using data collected at follow-up visit. Abbreviations: B2, stage 2 for breast development; B3-4, stage 3–4 for breast development; PH2, stage 2 for pubic hair growth; PH3-4, stage 3–4 for pubic hair growth. All ages are centered on birth dates ± 6 months.
(TIF)

S1 Table Baseline characteristics of adolescent Bangladeshi girls (9–15 years) who were included in the analysis in comparison to those lost to follow-up. Abbreviations: BMI, body mass index calculated as weight (kg) / height (m)^2^; BMIZ, body mass index-for-age z-score [33]; HAZ, height-for-age z-score [33]; SD, standard deviation. ^1^ Eligibility defined as girls who were alive, met, gave consent and agreed to interview.
(DOCX)

S2 Table Ages at which different stages of secondary sex characteristics and menarche were attained using data collected at baseline visit. Abbreviations: B2, stage 2 for breast development; B3-4, stage 3–4 for breast development; CI, confidence interval; P_3-97_, 3^rd^-97^th^ percentile, respectively; PH2, stage 2 for pubic hair growth; PH3-4, stage 3–4 for pubic hair growth. ^1^ Ages at attainment were estimated from probit analysis using baseline assessment at which adolescent girls were aged 9–15 years.
(DOCX)

S3 Table Ages at which different stages of secondary sex characteristics and menarche were attained using data collected at follow-up visit. Abbreviations: B2, stage 2 for breast development; B3-4, stage 3–4 for breast development; CI, confidence interval; P_3-97_, 3^rd^-97^th^ percentile, respectively; PH2, stage 2 for pubic hair growth; PH3-4, stage 3–4 for pubic hair growth. ^1^ Ages at attainment were estimated from probit analysis using follow-up assessment at which adolescent girls were aged 10–16 years.
(DOCX)

## Abbreviations

AA: African American
AAM: age at menarche
B1-5: each level of Tanner stage for breast development
CI: confidence interval
HSES: high socioeconomic status
LMIC: low and middle income country
LSES: low socioeconomic status
MA: Mexican American
NHW: non-Hispanic white
P_3-97_: 3^rd^-97^th^ percentile, respectively
PDS: pubertal development scale
PH1-5: each level of Tanner stage for pubic hair growth
SD: standard deviation
