# Supplementary figures and images for: Characterization of pubertal development of girls in rural Bangladesh
Source: PLoS One. 2021 Apr 2;16(4):e0247762. doi: 10.1371/journal.pone.0247762 (PMC8018666; doi:10.1371/journal.pone.0247762)

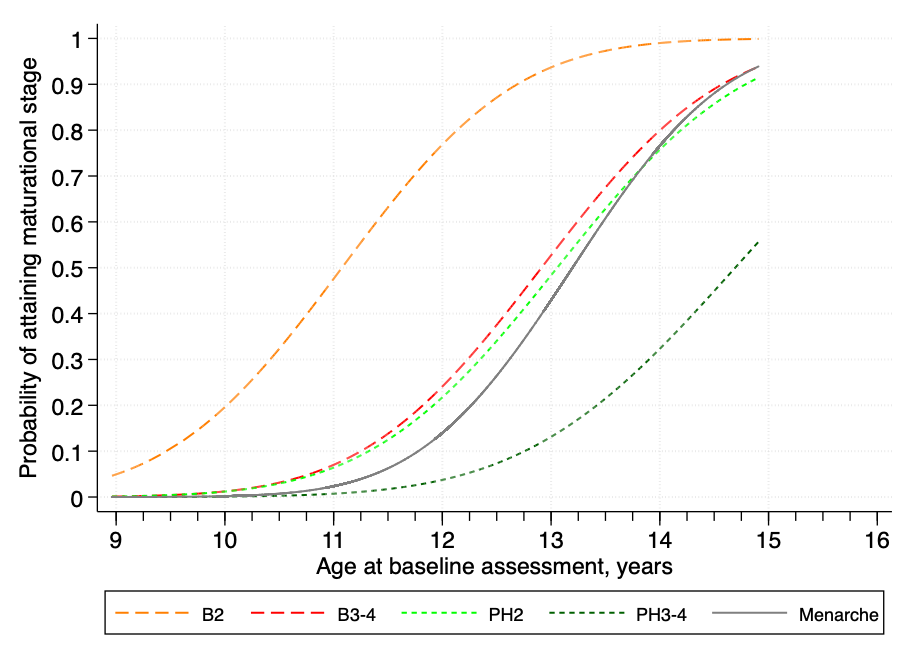

Supplement: S1 Fig — Abbreviations: B2, stage 2 for breast development; B3-4, stage 3–4 for breast development; PH2, stage 2 for pubic hair growth; PH3-4, stage 3–4 for pubic hair growth. All ages are centered on birth dates ± 6 months. (TIF) [file pone.0247762.s002.tif]

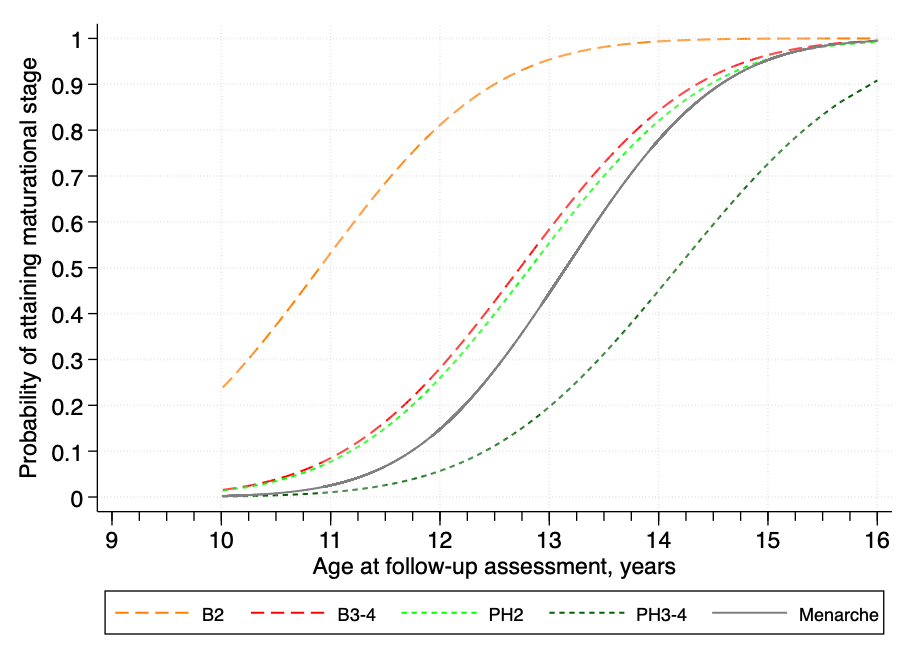

Supplement: S2 Fig — Abbreviations: B2, stage 2 for breast development; B3-4, stage 3–4 for breast development; PH2, stage 2 for pubic hair growth; PH3-4, stage 3–4 for pubic hair growth. All ages are centered on birth dates ± 6 months. (TIF) [file pone.0247762.s003.tif]
