# Supplementary material for: Characterization of pubertal development of girls in rural Bangladesh
Source: PLoS One. 2021 Apr 2;16(4):e0247762. doi: 10.1371/journal.pone.0247762 (PMC8018666; doi:10.1371/journal.pone.0247762)
Supplement: S1 Table — Abbreviations: BMI, body mass index calculated as weight (kg) / height (m)2; BMIZ, body mass index-for-age z-score [33]; HAZ, height-for-age z-score [33]; SD, standard deviation. 1 Eligibility defined as girls who were alive, met, gave consent and agreed to interview. (DOCX) [file pone.0247762.s004.docx]

| **S1 Table. Baseline characteristics of adolescent Bangladeshi girls (9-15 years) who were included in the analysis in comparison to those lost to follow-up.** | | | | | | | | | |
| --- | --- | --- | --- | --- | --- | --- | --- | --- | --- |
|  | Eligible^1^ at baseline | | | Eligible^1^ at follow-up | | | Lost to follow-up | | |
| Characteristic | *N* | *n* (%) | Mean ± SD | *N* | *n* (%) | Mean ± SD | *N* | *n* (%) | Mean ± SD |
| Age, years | 15,320 |  | 12.0 ± 1.3 | 14,057 |  | 11.9 ± 1.3 | 1,263 |  | 12.3 ± 1.3 |
| Height-for-age z-scores | 15,282 |  | -1.7 ± 1.0 | 14,023 |  | -1.7 ± 1.0 | 1,259 |  | -1.7 ± 1.0 |
| Short stature (HAZ <-2) |  | 5,850 (38.3) |  |  | 5,410 (38.6) |  |  | 440 (35.0) |  |
| BMI-for-age z-scores | 15,282 |  | -1.3 ± 1.1 | 14,023 |  | -1.3 ± 1.1 | 1,259 |  | -1.1 ± 1.1 |
| Thinness (BMIZ <-2) |  | 3,898 (25.5) |  |  | 3,637 (25.9) |  |  | 261 (20.7) |  |
| Currently enrolled in school | 15,160 | 14,603 (96.3) |  | 13,922 | 13,510 (97.0) |  | 1,238 | 1,093 (88.3) |  |
| Household asset ownership | 15,317 |  |  | 14,055 |  |  | 1,262 |  |  |
| Electricity |  | 6,371 (41.6) |  |  | 5,851 (41.6) |  |  | 520 (41.2) |  |
| Cycles |  | 7,791 (50.9) |  |  | 7,286 (51.8) |  |  | 505 (40.0) |  |
| Motorcycles |  | 1,110 (7.3) |  |  | 991 (7.1) |  |  | 119 (9.4) |  |
| Cell phones |  | 13,734 (89.7) |  |  | 12,600 (89.7) |  |  | 1,134 (89.9) |  |
| TV |  | 3,242 (21.2) |  |  | 2,943 (20.9) |  |  | 299 (23.7) |  |
| PC |  | 170 (1.1) |  |  | 155 (1.1) |  |  | 15 (1.2) |  |
| Abbreviations: BMI, body mass index calculated as weight (kg) / height (m)^2^; BMIZ, body mass index-for-age z-score [33]; HAZ, height-for-age z-score [33]; SD, standard deviation  ^1^ Eligibility defined as girls who were alive, met, gave consent and agreed to interview. | | | | | | | | | |
