# Supplementary material for: Characterization of pubertal development of girls in rural Bangladesh
Source: PLoS One. 2021 Apr 2;16(4):e0247762. doi: 10.1371/journal.pone.0247762 (PMC8018666; doi:10.1371/journal.pone.0247762)
Supplement: S2 Table — Abbreviations: B2, stage 2 for breast development; B3-4, stage 3–4 for breast development; CI, confidence interval; P3-97, 3rd-97th percentile, respectively; PH2, stage 2 for pubic hair growth; PH3-4, stage 3–4 for pubic hair growth. 1 Ages at attainment were estimated from probit analysis using baseline assessment at which adolescent girls were aged 9–15 years. (DOCX) [file pone.0247762.s005.docx]

| **S2 Table. Ages at which different stages of secondary sex characteristics and menarche were attained using data collected at baseline visit.^1^** | | | | | | | |
| --- | --- | --- | --- | --- | --- | --- | --- |
| Maturation | Age, years | | | | | | |
|  | P_3_ | P_10_ | P_25_ | P_50_ (95% CI) | P_75_ | P_90_ | P_97_ |
| Breast development, stage |  |  |  |  |  |  |  |
| B2 | 8.71 | 9.46 | 10.23 | 11.08 (11.05-11.10) | 11.93 | 12.69 | 13.44 |
| B3-4 | 10.47 | 11.25 | 12.04 | 12.91 (12.89-12.94) | 13.79 | 14.57 | 15.35 |
| Pubic hair growth, stage |  |  |  |  |  |  |  |
| PH2 | 10.51 | 11.32 | 12.14 | 13.06 (13.03-13.08) | 13.97 | 14.79 | 15.60 |
| PH3-4 | 11.85 | 12.76 | 13.67 | 14.69 (14.66-14.73) | 15.71 | 16.63 | 17.54 |
| Menarche | 11.11 | 11.78 | 12.45 | 13.20 (13.17-13.23) | 13.94 | 14.62 | 15.28 |
| Abbreviations: B2, stage 2 for breast development; B3-4, stage 3-4 for breast development; CI, confidence interval; P_3-97_, 3^rd^-97^th^ percentile, respectively; PH2, stage 2 for pubic hair growth; PH3-4, stage 3-4 for pubic hair growth  ^1^ Ages at attainment were estimated from probit analysis using baseline assessment at which adolescent girls were aged 9-15 years. | | | | | | | |
