# Supplementary material for: Characterization of pubertal development of girls in rural Bangladesh
Source: PLoS One. 2021 Apr 2;16(4):e0247762. doi: 10.1371/journal.pone.0247762 (PMC8018666; doi:10.1371/journal.pone.0247762)
Supplement: S3 Table — Abbreviations: B2, stage 2 for breast development; B3-4, stage 3–4 for breast development; CI, confidence interval; P3-97, 3rd-97th percentile, respectively; PH2, stage 2 for pubic hair growth; PH3-4, stage 3–4 for pubic hair growth. 1 Ages at attainment were estimated from probit analysis using follow-up assessment at which adolescent girls were aged 10–16 years. (DOCX) [file pone.0247762.s006.docx]

| **S3 Table. Ages at which different stages of secondary sex characteristics and menarche were attained using data collected at follow-up visit.^1^** | | | | | | | |
| --- | --- | --- | --- | --- | --- | --- | --- |
| Maturation | Age, years | | | | | | |
|  | P_3_ | P_10_ | P_25_ | P_50_ (95% CI) | P_75_ | P_90_ | P_97_ |
| Breast development, stage |  |  |  |  |  |  |  |
| B2 | 8.55 | 9.30 | 10.06 | 10.90 (10.86-10.93) | 11.74 | 12.50 | 13.25 |
| B3-4 | 10.36 | 11.11 | 11.88 | 12.73 (12.71-12.76) | 13.58 | 14.35 | 15.11 |
| Pubic hair growth, stage |  |  |  |  |  |  |  |
| PH2 | 10.41 | 11.18 | 11.96 | 12.83 (12.80-12.85) | 13.69 | 14.47 | 15.24 |
| PH3-4 | 11.59 | 12.41 | 13.25 | 14.17 (14.15-14.20) | 15.10 | 15.93 | 16.76 |
| Menarche | 11.08 | 11.74 | 12.41 | 13.15 (13.13-13.18) | 13.90 | 14.57 | 15.23 |
| Abbreviations: B2, stage 2 for breast development; B3-4, stage 3-4 for breast development; CI, confidence interval; P_3-97_, 3^rd^-97^th^ percentile, respectively; PH2, stage 2 for pubic hair growth; PH3-4, stage 3-4 for pubic hair growth  ^1^ Ages at attainment were estimated from probit analysis using follow-up assessment at which adolescent girls were aged 10-16 years. | | | | | | | |
